# Determining optimal conformity index values in pelvic region radiotherapy planning under ICRU 50/62, ICRU 83, and RTOG/NRG guidelines

**DOI:** 10.1002/acm2.70635

**Published:** 2026-05-24

**Authors:** Levent Gonultas

**Affiliations:** ^1^ Vocational School of Health Services Ankara Yıldırım Beyazıt University Ankara Türkiye; ^2^ Universal Radiotherapy R&D Co. Ltd. Ankara Türkiye

**Keywords:** conformity index (CI), unconformity index (UCIu, UCIo), radiotherapy plan evaluation, dose coverage and dose spill, quantitative plan assessment

## Abstract

**Background:**

Conformity evaluation is essential for assessing radiotherapy plan quality, yet widely used indices such as the ICRU and Paddick Conformity Index do not distinguish between target underdosage and peripheral overdose. As clinical protocols differ in their target coverage definitions ICRU 50/62, ICRU 83, and RTOG/NRG direct comparisons of plan conformity across protocols remain challenging. Recently proposed metrics the Universal Conformity Index (CI) and Unconformity Indices (UCIu for underdose and UCIo for overdose) provide a decomposed, interpretable assessment of dose target relationships. However, protocol‐specific optimal ranges for these indices and their clinical achievability have not been systematically established for pelvic radiotherapy.

**Purpose:**

To determine protocol‐specific optimal (p10–p90) ranges for CI, UCIu, and UCIo across pelvic sites under ICRU 50/62, ICRU 83, and RTOG/NRG frameworks, and to verify whether these optimal values are clinically achievable using modern IMRT plans.

**Methods:**

This study analyzed 40 anonymized pelvic cancer cases (20 prostate, 10 rectum, 10 endometrium). All cases were replanned under standardized dose prescriptions following ICRU 50/62, ICRU 83, and RTOG/NRG protocols. Two geometric configurations were evaluated: Target 1 (strict) and Target 2 (conservative). Stage 1: Using protocol‐specific V95(%) definitions, theoretical optimal CI, UCIu, and UCIo bands (p10–p90) were calculated separately for each site and protocol under Target 1 and Target 2. Stage 2: All 40 cases were combined into a pooled pelvic dataset to derive region‐wide optimal ranges and assess generalizability across sites. Stage 3: Clinical IMRT plans satisfying ICRU, RTOG, and QUANTEC constraints were generated for all patients, and clinical CI, UCIu, and UCIo values were compared against theoretical optimal bands. Statistical analysis included percentile bands, 95% Confidence Interval, p value, and effect size (*η* ^2^).

**Results:**

Across all pelvic sites, CI values decreased progressively from ICRU 50/62 to ICRU 83 and RTOG/NRG (*p* < 0.001), while UCIu and UCIo increased systematically, reflecting protocol‐driven differences in coverage tolerance. Margin expansion from Target 1 to Target 2 further reduced CI and increased UCIo. Pooled pelvic optimal ranges showed tight reproducibility with narrow 95% confidence intervals (± 0.006–0.016). Strong protocol effects were observed for CI (*η* ^2^ = 0.259–0.519), UCIu (*η* ^2^ = 0.997), and UCIo (*η* ^2^ up to 0.182). Clinically generated IMRT plans demonstrated high‐quality target coverage (V95% ≥ 98.9%) and acceptable OAR doses. Clinical CI, UCIu, and UCIo values closely matched the optimal p10–p90 bands. Prostate and rectum plans aligned fully with all protocol‐specific optimal ranges, while endometrium plans matched RTOG/NRG and Target‐2 thresholds, with minor deviations under strict Target‐1 geometry.

**Conclusions:**

This study provides the protocol‐specific and clinically achievable optimal ranges for CI, UCIu, and UCIo for pelvic radiotherapy across ICRU 50/62, ICRU 83, and RTOG/NRG frameworks. The combined theoretical, pooled, and clinical validation approach demonstrates that these decomposed conformity indices are robust, reproducible, and directly applicable to routine IMRT plan evaluation. The resulting optimal bands offer standardized benchmarks for assessing dose conformity and dose spill, improving cross‐protocol comparability and supporting future development of quantitative conformity‐based guidelines in radiotherapy.

## 1 INTRODUCTION

Radiotherapy is one of the most precise cancer treatment modalities, aiming to deliver the prescribed radiation dose to the tumor with optimal homogeneity, while minimizing exposure to surrounding normal tissues. The ability to objectively evaluate the quality of treatment plans has therefore become a cornerstone of modern radiation oncology. Over the last three decades, the International Commission on Radiation Units and Measurements (ICRU) has played a central role in establishing standards for prescribing, recording, and reporting radiotherapy. ICRU Report 50 (1993) introduced the fundamental concepts of gross tumor volume (GTV), clinical target volume (CTV), and planning target volume (PTV), ensuring consistency in target definition (ICRU, 1993). ICRU Report 62 (1999) expanded on these principles by incorporating setup and internal margins (ICRU, 1999),^1^ while ICRU Report 83 (2010) addressed the needs of intensity‐modulated radiotherapy (IMRT), emphasizing dose–volume histograms (DVHs) and quantitative indices as essential plan evaluation tools (ICRU, 2010).^2^ In parallel, the Radiation Therapy Oncology Group (RTOG) have translated these standards into protocol based criteria for clinical trials, defining explicit coverage, homogeneity, and normal tissue constraints that serve as benchmarks for plan quality and inter‐institutional consistency.^3^, ^4^, ^5^, ^6^, ^7^

Among the most widely applied metrics are the coverage index (COVI), the conformity index (CI), and the Homogeneity Index (HI). The COVI is defined as the proportion of the target volume (TV) receiving the prescribed dose to the TV:

(1) $$\mathrm{COVI}=\frac{V_{T,\;ref}}{V_{T}},\;$$

where, *V_T_* and *V_T_*_,_ *_ref_* are the TV and the TV encompassed by the reference isodose volume, respectively.

This simple ratio provides a direct measure of whether the prescribed dose adequately encompasses the planning tumor volume (PTV). However, while coverage is necessary, it is not sufficient for comprehensive plan evaluation because it does not account for irradiation of healthy tissues. CI is a measuring of how well prescription isodose volume (PIV) conforms to the size and shape of the tumor volume (TV) and healthy tissue volume (HTV). The CI should consider the negative effects on conformity of cold spots in the TV and HTV irradiated around the TV.^8^ The ICRU CI is defined as the ratio of the TV covered by the prescription isodose to the total PIV.

(2) $$\mathrm{CI}=\frac{V_{T,\;ref}}{V_{ref}}\;,$$

where, V_ref_ is the reference isodose volume.

The ICRU CI provides accurate results if the tumor volume is entirely encompassed by the PIV. But this index cannot distinguish between underdosage and excessive irradiation of normal tissues. Paddick (2000) introduced a modified CI:^9^

(3) $$\mathrm{CI}=\frac{V_{T,\;ref}}{V_{T}}\;\times \;\frac{V_{T,\;ref}}{V_{ref}}=\frac{{V_{T,\;ref}}^{2}}{V_{T}\times V_{ref}}\;$$

The first fraction of Paddick CI equation defines the quality of coverage of the tumor, the second fraction defines the volume of healthy tissue receiving a dose greater than or equal to the prescribed reference dose. But this CI remains insufficient when both the TV and the healthy tissues are only partially irradiated. In addition, these CI formulations are not universal in applicability and fail to quantify the magnitude and source of unconformity.^8^, ^10^To overcome these limitations, novel evaluation tools have recently been proposed. Universal CI and Unconformity Indices (UCIs) algorithms, which not only quantify the degree of conformity but also identify the magnitude and source of unconformity were introduced as follows.^10^, ^11^, ^12^, ^13^, ^14^

(4) $$\begin{array}{cc} \mathrm{CI} & =\frac{V_{T,\mathrm{ref}}}{V_{T}+V_{\mathrm{ref}}-V_{T,\;\mathrm{ref}}}\\ \mathrm{UC}I_{\mathrm{underdose}} & =\frac{V_{T}-V_{T,\;\mathrm{ref}}}{V_{T}+V_{\mathrm{ref}}-V_{T,\;\mathrm{ref}}}\\ \mathrm{UC}I_{\mathrm{overdose}} & =\frac{V_{\mathrm{ref}}-V_{T,\;\mathrm{ref}}}{V_{T}+V_{\mathrm{ref}}-V_{T,\;\mathrm{ref}}}, \end{array}$$

where, the CI calculates the conformity of dose distribution, and UCIs calculate the unconformity of dose distribution. So, the universal UCI_overdose_ and UCI_underdose_ equations reflect the negative effect of dose distribution, and universal CI reflects the positive effect of dose distribution. UCI_underdose_ and UCI_overdose_ calculate the negative effects on the dose conformity of partial irradiated TV and of irradiated healthy tissues, respectively.

While ICRU and RTOG/NRG metrics, as well as commonly used indices such as COVI and Paddick CI, remain essential for standardization and comparability, these approaches typically provide a single‐value summary of conformity and do not explicitly distinguish between underdose and overdose contributions. In contrast, the proposed UCI‐based framework enables a decomposed and geometry‐aware evaluation of conformity, allowing the magnitude and source of unconformity to be quantified separately. This work aims to establish benchmark values that can serve as references for optimal radiotherapy planning using these extended conformity metrics.

## 2 METHODS

### 2.1 Patient selection and data preparation

**FIGURE 1:**
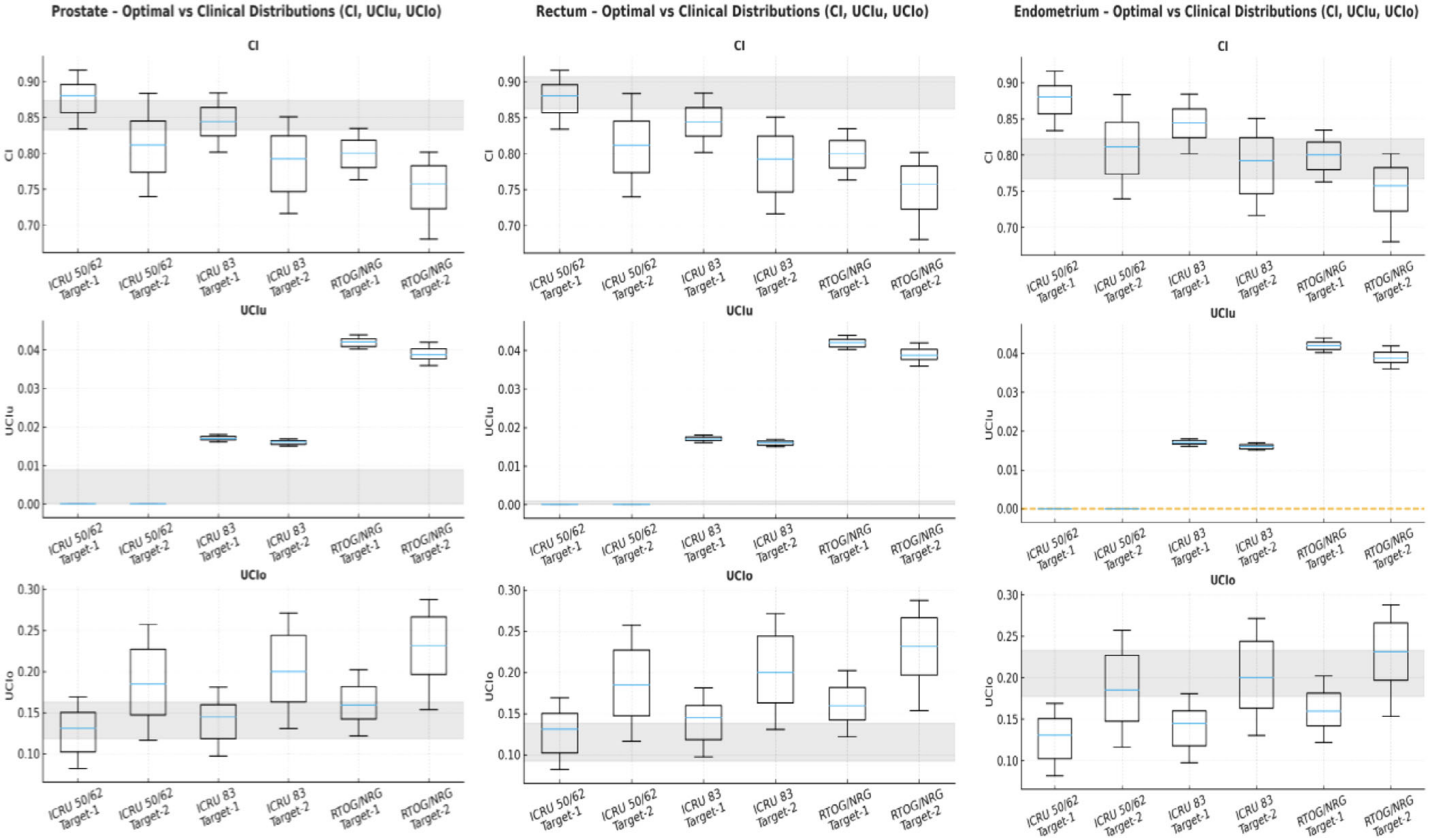
Each panel shows Target‐1 and Target‐2 Optimal p10–p90 box plots across the three protocols (ICRU 50/62, ICRU 83, RTOG/NRG). The shaded gray region indicates the Clinical p10–p90 range for each metric (CI, UCIu, UCIo).

This study analyzed external beam radiotherapy (EBRT) data from 40 pelvic cancer patients (20 prostate, 10 endometrium, 10 rectum). All cases were anonymized and replanned under standardized dose prescriptions following ICRU 50/62, ICRU 83, and RTOG/NRG guidelines. Two geometric configurations were evaluated: Target 1 (strict margin) and Target 2 (conservative margin). The methodology consisted of three stages: first, theoretical optimal CI, UCIu (UCI_underdose_) and UCIo (UCI_overdose_) ranges were calculated separately for each pelvic site and protocol under both geometric configurations (Table 1); second, all cases were combined into a pooled pelvic dataset to derive region‐wide optimal ranges and assess whether site‐specific values could be generalized across the pelvic region (considering prostate, endometrial, and rectal anatomical volumes as commonly defined in pelvic radiotherapy planning) (Table 2, 3); and finally, new treatment plans meeting ICRU, RTOG, and QUANTEC dose constraints were generated for all patients, and the resulting CI, UCIu, and UCIo values were compared with the theoretical optimal ranges to verify their clinical feasibility(Table 4, 5 and Figure 1).

**TABLE 1:** Mean ± SD and optimal values of CI, UCIu and UCIo (p10‐p90) and 95% confidence intervals values for target 1 and target 2 across ICRU50/62, ICRU83, and RTOG/NRG protocols in prostate, rectum, and endometrium cases. (Prostate PTV = 59,1cc–175cc, rectum PTV = 465,7cc–1092,2cc and endometrium PTV = 140,6cc–256,1cc).

			Target 1			Target 2		
Site	Metric	Protocol	Mean ± SD	Optimal Value (P10–P90)	95% Confidence Interval	Mean ± SD	Optimal Value (P10–P90)	95% Confidence Interval
Prostate	CI	ICRU50/62	0.852 ± 0.018	0.826–0.877	±0.008	0.755 ± 0.026	0.707–0.796	±0.012
		ICRU83	0.821 ± 0.017	0.797–0.845	±0.008	0.729 ± 0.025	0.683–0.768	±0.012
		RTOG/NRG	0.776 ± 0.015	0.754–0.798	±0.007	0.692 ± 0.023	0.649–0.728	±0.011
	UCIu	ICRU50/62	0.000 ± 0.000	0.000–0.000	±0.000	0.000 ± 0.000	0.000–0.000	±0.000
		ICRU83	0.017 ± 0.001	0.016–0.017	±0.000	0.015 ± 0.001	0.014–0.016	±0.000
		RTOG/NRG	0.041 ± 0.001	0.040–0.042	±0.000	0.036 ± 0.001	0.034–0.038	±0.001
	UCIo	ICRU50/62	0.148 ± 0.018	0.123–0.174	±0.008	0.245 ± 0.026	0.204–0.293	±0.012
		ICRU83	0.163 ± 0.017	0.138–0.187	±0.008	0.256 ± 0.025	0.216–0.303	±0.012
		RTOG/NRG	0.183 ± 0.016	0.160–0.206	±0.008	0.272 ± 0.024	0.234–0.317	±0.011
Rectum	CI	ICRU50/62	0.914 ± 0.009	0.899–0.926	±0.007	0.865 ± 0.023	0.826–0.887	±0.017
		ICRU83	0.880 ± 0.009	0.866–0.891	±0.006	0.834 ± 0.022	0.796–0.854	±0.016
		RTOG/NRG	0.831 ± 0.008	0.818–0.841	±0.006	0.788 ± 0.020	0.754–0.807	±0.014
	UCIu	ICRU50/62	0.000 ± 0.000	0.000–0.000	±0.000	0.000 ± 0.000	0.000–0.000	±0.000
		ICRU83	0.018 ± 0.000	0.017–0.018	±0.000	0.017 ± 0.000	0.016–0.017	±0.000
		RTOG/NRG	0.044 ± 0.000	0.043–0.044	±0.000	0.041 ± 0.001	0.040–0.042	±0.001
	UCIo	ICRU50/62	0.086 ± 0.009	0.074–0.101	±0.007	0.135 ± 0.023	0.113–0.174	±0.017
		ICRU83	0.102 ± 0.009	0.091–0.117	±0.006	0.149 ± 0.022	0.128–0.187	±0.016
		RTOG/NRG	0.126 ± 0.008	0.115–0.139	±0.006	0.171 ± 0.021	0.151–0.207	±0.015
Endometrium	CI	ICRU50/62	0.839 ± 0.010	0.823–0.856	±0.007	0.785 ± 0.015	0.759–0.800	±0.011
		ICRU83	0.809 ± 0.009	0.793–0.824	±0.007	0.757 ± 0.014	0.733–0.771	±0.010
		RTOG/NRG	0.765 ± 0.009	0.751–0.779	±0.006	0.717 ± 0.013	0.695–0.731	±0.009
	UCIu	ICRU50/62	0.000 ± 0.000	0.000–0.000	±0.000	0.000 ± 0.000	0.000–0.000	±0.000
		ICRU83	0.017 ± 0.000	0.016–0.017	±0.000	0.015 ± 0.000	0.015–0.016	±0.000
		RTOG/NRG	0.040 ± 0.000	0.040–0.041	±0.000	0.038 ± 0.001	0.037–0.038	±0.000
	UCIo	ICRU50/62	0.161 ± 0.010	0.144–0.177	±0.007	0.215 ± 0.015	0.200–0.241	±0.011
		ICRU83	0.175 ± 0.010	0.159–0.191	±0.007	0.227 ± 0.014	0.213–0.252	±0.010
		RTOG/NRG	0.191 ± 0.009	0.180–0.210	±0.007	0.245 ± 0.014	0.231–0.269	±0.010

**TABLE 2:** Comparison of CI, UCIu, and UCIo values for target 1 among protocols (ICRU50/62, ICRU83, RTOG/NRG) for pelvis region (*n* = 40; prostate + rectum + endometrium cases).

Metric	Protocol	Mean ± SD	Optimal Value (P10–P90)	95% Confidence Interval	*p*	*η* ^2^
CI	ICRU50/62	0.864 ± 0.033	0.831–0.919	±0.010	<0.001	0.519
	ICRU83	0.832 ± 0.031	0.801–0.885	±0.010		
	RTOG/NRG	0.787 ± 0.029	0.758–0.835	±0.009		
UCIu	ICRU50/62	0.000 ± 0.000	0.000–0.000	±0.000	<0.001	0.997
	ICRU83	0.017 ± 0.001	0.016–0.018	±0.000		
	RTOG/NRG	0.041 ± 0.002	0.040–0.044	±0.000		
UCIo	ICRU50/62	0.1356 ± 0.033	0.081–0.169	±0.010	<0.001	0.182
	ICRU83	0.1501 ± 0.032	0.097–0.183	±0.010		
	RTOG/NRG	0.172 ± 0.030	0.121–0.203	±0.010		

**TABLE 3:** Comparison of CI, UCIu, and UCIo values for target 2 among protocols (ICRU50/62, ICRU83, RTOG/NRG) for Pelvis Region (*n* = 40; prostate + rectum + endometrium cases).

Metric	Protocol	Mean ± SD	Optimal Value (P10–P90)	95% Confidence Interval	*p*	*η* ^2^
CI	ICRU50/62	0.790 ± 0.051	0.739–0.885	±0.016	<0.001	0.259
	ICRU83	0.762 ± 0.048	0.713–0.852	±0.015		
	RTOG/NRG	0.722 ± 0.045	0.677–0.805	±0.016		
UCIu	ICRU50/62	0.000 ± 0.000	0.000–0.000	±0.000	<0.001	0.991
	ICRU83	0.016 ± 0.001	0.015–0.017	±0.000		
	RTOG/NRG	0.038 ± 0.002	0.036–0.042	±0.001		
UCIo	ICRU50/62	0.210 ± 0.051	0.115–0.261	±0.016	0.025	0.061
	ICRU83	0.222 ± 0.049	0.130–0.272	±0.016		
	RTOG/NRG	0.240 ± 0.047	0.152–0.288	±0.015		

**TABLE 4:** Dosimetric parameters and conformity indices for clinical IMRT plans of prostate, rectum, and endometrium cases.

Structures	Parameters	Prostate (Mean ± SD)	Prostate (p10–p90)	Rectum (Mean ± SD)	Rectum (p10–p90)	Endometrium (Mean ± SD)	Endometrium (p10–p90)
**PTV1**	V95(%)	99.73 ± 0.46	98.92–100.00	100.00 ± 0.00	100.00–100.00	100.00 ± 0.00	100.00–100.00
	V100(%)	93.96 ± 2.28	90.00–97.38	87.33 ± 3.44	82.00–90.70	84.24 ± 3.15	79.00–87.30
	Dmax(%)	106.44 ± 0.75	105.40–107.64	107.10 ± 0.18	106.80–107.40	106.82 ± 0.16	106.60–107.00
	D98(%)	98.63 ± 1.00	96.82–99.74	98.40 ± 0.28	97.90–98.70	98.40 ± 0.16	98.20–98.60
	D2(%)	105.06 ± 0.43	104.42–105.82	105.34 ± 0.16	105.00–105.50	105.44 ± 0.68	104.90–106.60
	CI(95%)	0.859 ± 0.014	0.833–0.874	0.884 ± 0.020	0.862–0.907	0.800 ± 0.021	0.767–0.823
	UCIu(95%)	0.002 ± 0.004	0.000–0.009	0.000 ± 0.000	0.000–0.001	0.000 ± 0.000	0.000–0.000
	UCIo(95%)	0.139 ± 0.016	0.118–0.163	0.116 ± 0.020	0.092–0.138	0.200 ± 0.021	0.177–0.233
**PTV2**	V95(%)	99.94 ± 0.10	99.76–100.00	99.86 ± 0.20	99.50–100.00	99.88 ± 0.16	99.70–100.00
	V100(%)	94.17 ± 2.30	89.18–96.38	77.03 ± 5.06	69.70–83.40	54.42 ± 5.97	46.40–60.20
	D98(%)	98.10 ± 0.45	97.53–98.91	96.44 ± 0.30	95.90–96.91	96.44 ± 0.21	96.24–96.78
**Rectum**	V50Gy(%)	22.75 ± 5.43	13.94–30.70	–	–	10.86 ± 5.55	2.60–6.40
	V60Gy(%)	11.15 ± 3.91	5.30–16.20	–	–	0.00 ± 0.00	0.00–0.00
	V65Gy(%)	8.48 ± 3.35	3.32–12.82	–	–	0.00 ± 0.00	0.00–0.00
	V70Gy(%)	6.05 ± 2.75	1.86–9.68	–	–	0.00 ± 0.00	0.00–0.00
	V75Gy(%)	2.39 ± 1.36	0.36–4.16	–	–	0.00 ± 0.00	0.00–0.00
**Bladder**	V65Gy(%)	6.71 ± 4.35	3.00–14.60	0.00 ± 0.00	0.00–0.00	0.00 ± 0.00	0.00–0.00
	V70Gy(%)	5.53 ± 4.00	2.34–12.68	0.00 ± 0.00	0.00–0.00	0.00 ± 0.00	0.00–0.00
	V75Gy(%)	3.39 ± 2.66	1.28–8.36	0.00 ± 0.00	0.00–0.00	0.00 ± 0.00	0.00–0.00
	V80Gy(%)	0.00 ± 0.00	0.00–0.00	0.00 ± 0.00	0.00–0.00	0.00 ± 0.00	0.00–0.00
**Femoral head R**	Dmax(Gy)	29.64 ± 3.59	25.87–36.81	43.04 ± 1.38	40.68–44.91	43.52 ± 0.56	42.84–44.40
**Femoral head L**	Dmax(Gy)	29.92 ± 5.36	22.30–38.99	42.82 ± 1.90	39.34–45.48	42.32 ± 1.65	39.47–43.63
**Penile bulb**	Dmean(Gy)	29.47 ± 16.24	6.69–47.88	–	–	–	–
**Small bowel**	V45Gy(cc)	22.80 ± 37.99	0.00–107.00	32.91 ± 28.32	5.55–75.00	32.91 ± 28.32	5.55–75.00

**TABLE 5:** Comparison of Clinical and Optimal p10–P90 Ranges for Conformity and Unconformity Indexes (CI, UCIu, UCIo) Across protocols (ICRU50/62, ICRU83, RTOG/NRG).

Site	Metric	Clinical p10–p90	Protocol	Target‐1 Optimal p10‐p90	Target‐2 Optimal p10‐p90	Status Target‐1	Status Target‐2
**Prostate**	CI	0.833–0.874	ICRU 50/62	0.831–0.919	0.739–0.885	pass	pass
			ICRU 83	0.801–0.885	0.713–0.852	pass	pass
			RTOG/NRG	0.758–0.835	0.677–0.805	pass	pass
	UCIu	0.000–**0.009**	ICRU 50/62	0.000–0.000	0.000–0.000	fail	fail
			ICRU 83	0.016–0.018	0.015–0.017	pass	pass
			RTOG/NRG	0.040–0.044	0.036–0.042	pass	pass
	UCIo	0.118–0.163	ICRU 50/62	0.081–0.169	0.115–0.261	pass	pass
			ICRU 83	0.097–0.183	0.130–0.272	pass	pass
			RTOG/NRG	0.121–0.203	0.152–0.288	pass	pass
**Rectum**	CI	0.862–0.907	ICRU 50/62	0.831–0.919	0.739–0.885	pass	pass
			ICRU 83	0.801–0.885	0.713–0.852	pass	pass
			RTOG/NRG	0.758–0.835	0.677–0.805	pass	pass
	UCIu	0.000–**0.001**	ICRU 50/62	0.000–0.000	0.000–0.000	fail	fail
			ICRU 83	0.016–0.018	0.015–0.017	pass	pass
			RTOG/NRG	0.040–0.044	0.036–0.042	pass	pass
	UCIo	0.092–0.138	ICRU 50/62	0.081–0.169	0.115–0.261	pass	pass
			ICRU 83	0.097–0.183	0.130–0.272	pass	pass
			RTOG/NRG	0.121–0.203	0.152–0.288	pass	pass
**Endomet‐rium**	CI	0.767–0.823	ICRU 50/62	0.831–0.919	0.739–0.885	fail	pass
			ICRU 83	0.801–0.885	0.713–0.852	fail	pass
			RTOG/NRG	0.758–0.835	0.677–0.805	pass	pass
	UCIu	0.000–0.000	ICRU 50/62	0.000–0.000	0.000–0.000	pass	pass
			ICRU 83	0.016–0.018	0.015–0.017	pass	pass
			RTOG/NRG	0.040–0.044	0.036–0.042	pass	pass
	UCIo	0.177–0.233	ICRU 50/62	0.081–0.169	0.115–0.261	fail	pass
			ICRU 83	0.097–0.183	0.130–0.272	fail	pass
			RTOG/NRG	0.121–0.203	0.152–0.288	fail	pass

### 2.2 Treatment planning

Treatment planning was performed using the Eclipse Treatment Planning System (Varian Medical Systems, Palo Alto, CA, USA). For all cases, 6‐MV photon beams were delivered with a simultaneous integrated boost (SIB) IMRT technique employing seven coplanar fields. Dose calculations used the Anisotropic Analytical Algorithm (AAA). Two PTVs were defined for each disease site, with PTV2 constructed to encompass PTV1 (PTV2 ⊇ PTV1):

- Prostate: PTV1 included only the prostate gland and was prescribed 74 Gy (2 Gy × 37 fractions). PTV2 encompassed PTV1 plus the seminal vesicles (prostate + SV) and received an equivalent dose (56,25 Gy) of 54 Gy (2 Gy × 27 fractions) in 37 fractions.
- Rectum / Endometrium: PTV1 covered the GTV and the entire mesorectum or tumor bed, prescribed to 50.4 Gy (1.8 Gy × 28 fractions). PTV2 was generated by expanding PTV1 to additionally include the elective pelvic lymphatic regions and received an equivalent dose (45,6 Gy) of 45 Gy (1.8 Gy × 25 fractions) in 28 fractions.

### 2.3 Evaluation criteria

Plan quality was evaluated in accordance with the recommendations of ICRU Report 50, 62, 83, RTOG and QUANTEC dose constraints. For each patient, PTV coverage and organ‐at‐risk (OAR) doses were extracted and summarized in tabulated form. All treatment plans were designed to meet these established protocols, ensuring that PTV coverage was adequate while OAR doses remained within recommended limits. Reported parameters included V95%(%), V100%(%), Dmax(%), D98%(%), and D2%(%) for PTV as well as relevant dose‐volume metrics for OARs (e.g., Dmean, Dmax, and Vx).

### 2.4 Determination of optimal CI, UCIu, and UCIo constraint values

The acceptable limits of the CI and Unconformity Indexes (UCIu and UCIo) must be carefully evaluated when defining optimal plan quality in terms of plan conformity. An ideal CI algorithm should simultaneously assess two fundamental aspects of a dose distribution:

1. the conformity between the reference dose and the TV, and
2. the conformity between the reference dose and the surrounding healthy tissue.

In this context, ICRU 50–62, ICRU 83, and RTOG/NRG provide recommendations for the first aspect adequate target coverage by the reference dose:

- ICRU 50/62: V95% = 100% → the 95% isodose should completely cover the TV.
- ICRU 83: D98% ≥ 95% → at least 98% of the target should receive 95% of the prescribed dose.
- RTOG/NRG: V95% ≥ 95% → at least 95% of the target should receive 95% of the prescribed dose.

These criteria formed the foundation for defining acceptable CI‐related benchmark levels for adequate coverage. However, these reports do not provide explicit quantitative limits regarding how far the reference isodose (e.g., V95%) may extend beyond the PTV into surrounding healthy tissues. To define an acceptable upper bound for peripheral dose extension, the geometric outer margins were derived conceptually based on RTOG conformity criteria.

In ICRU 62 and ICRU 83^1^, ^2^ the CI is defined as CI = *V_ref_* / *V_T_*, which inherently quantifies the extent to which the reference isodose volume exceeds the TV. Historically, RTOG guidelines considered a CI range of 1.0–2.0 to be acceptable^6^; this was later refined, defining CI < 1.2 as acceptable and CI < 1.5 as a minor deviation.^7^

A CI value of 1.2 implies that the reference isodose volume may exceed the PTV volume by approximately 20%, while a CI of 1.5 corresponds to approximately 50% excess volume.

Therefore, an “acceptable” overdose‐related conformity deviation corresponds approximately to a 0.2 excess ratio relative to the PTV volume. This provides an objective geometric rationale for selecting the outer‐margin tolerances. Accordingly, two geometric outer‐margin targets were defined to represent different conformity tolerance levels:

- **Target 1 (higher conformity tolerance)**
  Outer margin (mm):
$$x_{1}=1,y_{1}=1,z_{1}=0;\;x_{2}=1,y_{2}=1,z_{2}=0$$
- **Target 2 (moderate conformity tolerance)**
  Outer margin (mm):
$$x_{1}=2,y_{1}=2,z_{1}=1;\;x_{2}=2,y_{2}=2,z_{2}=1,$$

where *x*_1_, *y*_1_, *z*_1_, *x*_2_, *y*_2_, and *z*_2_ represent the right lateral, anterior, superior, left lateral, posterior, and inferior directions, respectively.

They serve as geometric benchmark shells to bound the maximum acceptable extension of the reference isodose volume beyond the target, consistent with RTOG‐defined acceptable CI ranges. As shown in Table 1, a UCIo value of approximately 0.2 is achieved with these outer‐margin definitions, consistent with the acceptable CI deviation threshold derived from RTOG criteria. Target 1 corresponds to plans with higher conformity and improved healthy tissue sparing, whereas Target 2 represents a more relaxed but still clinically acceptable conformity band. These outer‐margin definitions do not represent patient‐specific clinical margins or setup uncertainty expansions. Rather, they were deliberately defined as standardized geometric shells to enable a consistent and reproducible framework for the spatial quantification of dose spillage and conformity behavior across different treatment plans.

This approach enables standardized comparative analysis, while patient‐specific anatomical variability is inherently reflected in the observed distribution of conformity metrics. In plans involving multiple PTVs at different dose levels, CI, UCIu, and UCIo should be evaluated separately for each PTV. If high‐ and low‐dose PTVs overlap, conformity assessment may be conceptually performed in non‐overlapping regions (e.g., subtracting the smaller PTV from the larger before applying the outer‐margin logic). To avoid ambiguity, in the present study optimal CI, UCIu, and UCIo constraint values were reported only for the high‐dose PTV.

### 2.5 Statistical analysis

The statistical analyses were performed to define the methodological framework used for determining optimal plan quality parameters in terms of dose conformity. Specifically, the analyses aimed to identify and justify the optimal constraint ranges of the CI and Unconformity Indices (UCIu for under and UCIo for over) under three different dose prescription protocols (ICRU50/62, ICRU 83, and RTOG/NRG). Each statistical parameter served a distinct methodological purpose:

- Mean ± Standard Deviation (SD) was used to describe the distribution and variability of CI, UCIu, and UCIo values within each dataset.
- The 10th–90th percentile (p10–p90) range was used to define the expected interval of optimal plan performance in terms of dose conformity by excluding the effect of outliers. In this study, the term “optimal” does not refer to outcome‐based optimality. Instead, it denotes a geometric benchmark band derived from: ICRU and RTOG/NRG target‐coverage recommendations (V95% criteria), and RTOG‐based acceptable conformity deviation limits reflected in the outer‐margin targets. The use of the p10–p90 interval allows for a robust representation of the central distribution while minimizing the influence of extreme values, thereby defining a clinically realistic and achievable conformity range in the presence of anatomical and planning variability.
- The 95% Confidence Interval was calculated with a precision threshold of ± 0.02, to ensure that the sample size for each dataset was statistically adequate for reliable constraint definition.
- The *p*‐value (p) obtained from one‐way ANOVA was used to test the presence of significant differences among protocols.
- Effect size (*η* ^2^) was included to quantify the magnitude and strength of observed differences.

These statistical parameters were collectively used to establish a consistent and reproducible framework for defining optimal CI, UCIu, and UCIo ranges for each protocol and anatomical site. All statistical operations were carried out using Microsoft Excel 2021 and verified using Python 3.11 libraries.

### 2.6 Interpretation of percentile‐based optimal bands

To facilitate clinical implementation, percentile‐based decision criteria were defined to link the calculated statistical ranges to plan‐evaluation thresholds:

Ideal reference condition: A perfect overlap between TV and PIV represents the theoretical upper performance limit (CI = 1, UCIu = 0, UCIo = 0).

Definition of optimal bands:

For CI: the p10–p90 interval represents the practical optimal conformity band; p10 serves as a conservative lower‐acceptance threshold. For UCIu and UCIo: the p90 percentile represents a conservative upper‐limit threshold for acceptable under‐ and over‐dose components.

Plan‐acceptance logic:

CI ≥ p10 → acceptable conformity; CI < p10 → suboptimal conformity (warning).

UCIu ≤ p90 → adequate coverage; UCIu > p90 → under‐coverage risk.

UCIo ≤ p90 → acceptable dose spill; UCIo > p90 → excessive spill (alert).

## 3 RESULTS

Using the percentile based (p10–p90) framework optimal CI, UCIu and UCIo ranges were determined for prostate, rectum, and endometrium under the ICRU 50/62, ICRU 83, and RTOG/NRG protocols (Table 1). Across all sites and protocols, the optimal CI values decreased systematically from ICRU 50/62 to ICRU 83 and RTOG/NRG, while both UCIu and UCIo increased progressively, reflecting the gradual relaxation of the V95% coverage definition. Similarly, expanding the outer‐margin tolerance from Target 1 (strict) to Target 2 (conservative) led to lower CI values and moderately higher UCIo, consistent with the geometric allowance for peripheral dose extension.

For prostate, CI decreased from 0.826–0.877 to 0.797–0.845 and 0.754–0.798 (Target 1) and from 0.707–0.796 to 0.683–0.768 and 0.649–0.728 (Target 2). UCIu increased from 0.000–0.000 to 0.016–0.017 and 0.040–0.042, while UCIo rose from 0.123–0.174 to 0.138–0.187 and 0.160–0.206 (Target 1), and similarly from 0.204–0.293 to 0.216–0.303 and 0.234–0.317 (Target 2). For rectum, CI decreased from 0.899–0.926 to 0.866–0.891 and 0.818–0.841 (Target 1) and from 0.826–0.887 to 0.796–0.854 and 0.754–0.807 (Target 2). UCIu increased from 0.000–0.000 to 0.017–0.018 and 0.043–0.044, while UCIo increased from 0.074–0.101 to 0.091–0.117 and 0.115–0.139 (Target 1), and from 0.113–0.174 to 0.128–0.187 and 0.151–0.207 (Target 2). For endometrium, CI decreased from 0.823–0.856 to 0.793–0.824 and 0.751–0.779 (Target 1) and from 0.759–0.800 to 0.733–0.771 and 0.695–0.731 (Target 2). UCIu increased from 0.000–0.000 to 0.016–0.017 and 0.040–0.041, while UCIo rose from 0.144–0.177 to 0.159–0.191 and 0.180–0.210 (Target 1), and from 0.200–0.241 to 0.213–0.252 and 0.231–0.269 (Target 2). These results collectively indicate that the defined optimal ranges are statistically reproducible (95% Confidence Intervals; ± 0.006–0.012) and clinically reliable, providing protocol and margin‐specific thresholds for acceptable conformity and dose uniformity in prostate, rectum, and endometrium radiotherapy planning.

Following the site specific analyses, all cases were aggregated into a pooled pelvic dataset to verify whether the optimal CI, UCIu, and UCIo values defined for individual sites could be generalized to the entire pelvic region. This analysis aimed to establish region wide optimal ranges under both geometric configurations (Target 1 and Target 2) and to determine whether the protocol dependent differences observed at the site level remained statistically significant when all pelvic cases were combined (Tables 2 and 3).

For the pooled pelvic dataset, the optimal CI (p10p90) demonstrated a significant protocol‐dependent decrease from 0.831–0.919 (ICRU 50/62) to 0.801–0.885 (ICRU 83) and 0.758–0.835 (RTOG/NRG) (*p* < 0.001, *η* ^2^ = 0.519), corresponding to a large effect size. The underdose component (UCIu) increased from 0.000–0.000 to 0.016–0.018 and 0.040 0.044, showing an extremely large effect size (*p* < 0.001, *η* ^2^ = 0.997). Similarly, the overdose component (UCIo) rose from 0.081–0.169 to 0.097–0.183 and 0.121–0.203, with a statistically significant difference (*p* < 0.001, *η* ^2^ = 0.182), corresponding to a large effect. The narrow 95% confidence intervals (± 0.009–0.010) confirm the reproducibility of these optimal ranges. When the outer‐margin tolerance expanded from Target 1 to Target 2, a similar trend was observed. The CI decreased significantly from 0.739–0.885 (ICRU 50/62) to 0.713–0.852 (ICRU 83) and 0.677–0.805 (RTOG/NRG) (*p* < 0.001, *η* ^2^ = 0.259), indicating a large effect. The underdose index (UCIu) increased from 0.000–0.000 to 0.015–0.017 and 0.036–0.042, again demonstrating an extremely large effect (*p* < 0.001, *η* ^2^ = 0.991). The overdose component (UCIo) also rose from 0.115–0.261 (ICRU 50/62) to 0.130–0.272 (ICRU 83) and 0.152–0.288 (RTOG/NRG), with a statistically meaningful difference (p = 0.025, *η* ^2^ = 0.061), corresponding to a moderate effect. Despite margin relaxation, the tight 95% confidence intervals (± 0.015–0.016) indicate high statistical reliability and low inter plan variability.

To determine whether the optimal p10–p90 ranges derived for CI, UCIu, and UCIo are achievable under real clinical conditions, a total of 40 IMRT plans were evaluated using protocol‐based PTV and OAR constraints (ICRU 50/62, ICRU 83, RTOG/NRG, QUANTEC). All clinical plans satisfied key PTV1 metrics V95% values of 98.9–100.0% (prostate), 100% (rectum), and 100% (endometrium), D98% ranges of 96.8–99.7%, and D2% values between 104.4% and 106.6% indicating consistent and protocol compliant dose coverage (Table 4). OAR doses also remained within recommended QUANTEC limits (e.g., rectum V60Gy = 5.3–16.2%, bladder V65Gy = 3.0–14.6%, femoral head Dmax < 45 Gy), confirming that all evaluated plans represent clinically acceptable high quality treatment deliveries.

The clinical conformity and unconformity indices closely matched the optimal p10–p90 ranges defined for each protocol. (Table 5) For prostate PTV1, clinical CI ranged from 0.833 to 0.874, overlapping the optimal Target‐1 bands for all protocols (ICRU 50/62: 0.831–0.919; ICRU 83: 0.801–0.885; RTOG/NRG: 0.758–0.835). Clinical UCIu (0.000–0.009) failed only the strict ICRU 50/62 optimal band (0.000–0.000) but met ICRU 83 (0.016–0.018) and RTOG/NRG (0.040–0.044) expectations. Clinical UCIo (0.118–0.163) fell fully within optimal bands across all three protocols (ICRU 50/62: 0.081–0.169; ICRU 83: 0.097–0.183; RTOG/NRG: 0.121–0.203). For rectum, clinical CI (0.862–0.907) was within the optimal protocol bands (ICRU 50/62: 0.831–0.919; ICRU 83: 0.801–0.885; RTOG/NRG: 0.758–0.835). Clinical UCIu (0.000–0.001) did not meet only ICRU 50/62 (0.000–0.000) but matched ICRU 83 (0.016–0.018) and RTOG/NRG (0.040–0.044). Clinical UCIo (0.092–0.138) was fully within optimal ranges for all protocols. For endometrium, a target size–dependent pattern was observed. For Target‐1, clinical CI (0.767–0.823) fell below the ICRU 50/62 and ICRU 83 optimal bands but aligned with RTOG/NRG (0.758–0.835). Clinical UCIu (0.000–0.000) satisfied all protocols, while UCIo (0.177–0.233) exceeded optimal ranges for ICRU 50/62 (0.081–0.169) and ICRU 83 (0.097–0.183) but matched RTOG/NRG (0.121–0.203). Under the broader Target‐2 definitions, endometrium clinical CI and UCIo values were within the optimal ranges for all protocols.

Figure 1 visually confirms these findings: the clinical p10–p90 bands (gray regions) overlap substantially with the optimal ranges for prostate and rectum and with the Target‐2 ranges for endometrium. This overlap demonstrates that the theoretically derived p10–p90 bands represent clinically realistic conformity and unconformity thresholds.

## 4 DISCUSSION

This study provides protocol specific optimal ranges (p10–p90) for the CI, UCIu and UCIo under the ICRU 50/62, ICRU 83, and RTOG/NRG guidelines.^1^, ^2^, ^3^, ^4^, ^5^, ^6^, ^7^, ^15^ Because clinical practice varies widely some centers follow ICRU 50/62 normalization, others adopt the D98% based ICRU 83 definitions, while many modern clinics use RTOG/NRG providing separate optimal thresholds for each guideline addresses an important gap in radiotherapy planning and evaluation. Previous studies have highlighted significant challenges in comparing plan conformity across institutions, largely due to limitations of existing conformity indices and inconsistencies in dose‐coverage definitions and normalization approaches. Together, these inconsistencies have been shown to hamper meaningful inter institutional comparison of plan quality, underscoring the need for protocol specific and decomposed conformity metrics such as CI, UCIu, and UCIo.^8^, ^9^, ^10^, ^16^, ^17^, ^18^, ^19^

A unique strength of this study is the use of two geometric target configurations designed to model strict versus relaxed V95% coverage tolerances. The relationship between geometric margins and high‐dose spread is well established in radiotherapy physics. van Herk described that increasing margins inherently expands the high‐dose region into surrounding normal tissues (“unavoidable dose bath increase”) as a direct geometric consequence of error compensation.^20^ Similarly, Stroom and Heijmen emphasized that geometric margin expansion inevitably increases irradiated normal‐tissue volume regardless of planning technique.^21^ These well‐known physical principles align directly with our findings: when transitioning from Target‐1 to Target‐2, CI systematically decreased and UCIo increased for all protocols, demonstrating the expected dose‐spill behavior under larger margins.

The importance of quantifying dose spill beyond the PTV is strongly supported by the literature. Peeters et al. reported that regions of unintended high‐dose spill outside the target are significant predictors of late rectal toxicity, even when DVH constraints are met.^22^ Hasselle et al. showed that extra‐PTV high‐dose exposure independently predicts gastrointestinal toxicity in gynecologic radiotherapy,^23^ while Rancati et al. described dose bath effects as key contributors to bowel morbidity.^24^ QUANTEC reports also highlight the contribution of high‐dose regions outside the PTV to gastrointestinal and genitourinary toxicity, emphasizing the need for minimizing hotspots and dose spill during plan optimization.^25^

Collectively, these findings validate the clinical importance of the overdose component captured by UCIo. Unlike traditional DVH‐based organ‐at‐risk limits, which evaluate dose to organs individually, UCIo captures the global 3D behavior of the PIV relative to the TV, providing a more comprehensive measure of unintended dose extension beyond the PTV.

Multiple RTOG protocols particularly in SBRT (e.g., RTOG 0813, 0915) explicitly classify plans as “acceptable,” “minor deviation,” or “major deviation” according to excess dose spillage outside the PTV.^7^ This concept aligns closely with the UCIo definition: both quantify the degree to which the high‐dose region extends into normal tissues. Therefore, UCIo serves as a generalized 3D conformity‐spill metric consistent with established RTOG quality‐assessment philosophies.

The evaluation of 40 clinical IMRT plans (20 prostate, 10 rectum, 10 endometrium) demonstrated that all plans met ICRU, RTOG, and QUANTEC requirements, confirming that they represent high‐quality clinical treatments. Importantly, the clinical CI, UCIu, and UCIo values closely matched the optimal p10–p90 ranges computed for each protocol. For prostate and rectum cases, the clinical conformity metrics showed strong agreement with the optimal ranges across all protocol definitions, including those aligned with RTOG‐based conformity expectations.^7^ This consistency further supports that the proposed optimal ranges are in line with established clinical benchmarks. Endometrium plans showed closer agreement with the RTOG and Target‐2 optimal bands, while deviations observed under stricter Target‐1 conditions can be interpreted as minor deviations within clinically acceptable tolerance ranges according to RTOG‐defined conformity criteria,^7^ suggesting that wider margins may better reflect clinically achievable dose‐spill characteristics in anatomically complex target geometries.

Figure 1 illustrates these relationships, showing a strong overlap between clinical performance and the theoretically derived optimal ranges. This study provides three key advancements to the field: Firstly, to our knowledge, no prior work has defined separate optimal conformity and unconformity ranges for ICRU 50/62, ICRU 83, and RTOG/NRG. This enables standardized evaluation of plan quality according to the protocol used. Secondly, the geometric relationship between coverage tolerance and dose spill is well established theoretically, but this work provides the first systematic numerical characterization of how strict versus relaxed V95% coverage conditions influence CI, UCIu, and UCIo. Finally, demonstrating that clinical IMRT plans naturally achieve the proposed optimal bands validates their practical applicability and supports their use as reference thresholds in routine plan evaluation.

## 5 CONCLUSIONS

This study established protocol‐specific optimal (p10–p90) ranges for CI, UCIu and UCIo under the ICRU 50/62, ICRU 83, and RTOG/NRG frameworks, providing a standardized and guideline‐compatible basis for evaluating dose conformity in pelvic radiotherapy planning. By decomposing dose conformity into under‐ and over‐dose components, the CI/UCIu/UCIo triplet offers a more interpretable representation of dose‐coverage behavior than classical conformity indices.

The analysis demonstrated that geometric margin expansion (Target‐1 → Target‐2) systematically reduces conformity and increases peripheral dose spill, in alignment with established radiotherapy physics principles. The tight 95% confidence intervals confirm the statistical reproducibility of the optimal ranges for all protocols and margin configurations.

Evaluation of 40 clinical IMRT plans further validated the clinical applicability of the proposed optimal bands. Clinical CI, UCIu, and UCIo values for prostate, rectum, and endometrium showed strong agreement with the protocol‐specific optimal ranges, particularly under ICRU 83 and RTOG/NRG definitions. Overall, the protocol‐specific optimal p10–p90 ranges derived in this study provide reliable and clinically achievable thresholds for assessing plan conformity and dose uniformity in pelvic radiotherapy. These results support the use of CI, UCIu, and UCIo as practical reference metrics for standardized, guideline‐consistent evaluation of radiotherapy treatment plans.

## AUTHOR CONTRIBUTIONS

Levent Gonultas conceived the study, developed the methodology, performed the analysis, and wrote the manuscript.

## CONFLICT OF INTEREST STATEMENT

The authors declare no conflicts of interest.

## ETHICS APPROVAL AND CONSENT TO PARTICIPATE

The study was approved by the Ankara Yıldırım Beyazıt University Ethics Committee (Approval Number: 03/1161) and conducted in accordance with the Declaration of Helsinki. This retrospective study utilized anonymized Computed Tomography (CT) datasets obtained from the radiation oncology archive. Due to the retrospective nature of the study and the use of anonymized data, the requirement for informed consent was waived by the Ethics Committee.

## Data Availability

The data that support the findings of this study are available from the corresponding author upon reasonable request.
